# Activation of inflammatory responses in human U937 macrophages by particulate matter collected from dairy farms: an in vitro expression analysis of pro-inflammatory markers

**DOI:** 10.1186/1476-069X-11-17

**Published:** 2012-03-28

**Authors:** Christoph FA Vogel, Johnny Garcia, Dalei Wu, Diane C Mitchell, Yanhong Zhang, Norman Y Kado, Patrick Wong, Danitza Alvizar Trujillo, Anna Lollies, Deborah Bennet, Marc B Schenker, Frank M Mitloehner

**Affiliations:** 1 Department of Environmental Toxicology, University of California, Davis, One Shields Avenue, Davis, CA 95616, USA; 2 Center for Health and The Environment, University of California, Davis, One Shields Avenue, Davis, CA 95616, USA; 3 Department of Public Health Sciences, University of California, Davis, One Shields Avenue, Davis, CA 95616, USA; 4 Department of Animal Science, University of California, Davis, One Shields Avenue, Davis, CA 95616, USA; 5 California Environmental Protection Agency, Air Resources Board, Sacramento, CA 95814, USA

**Keywords:** AhR, IL-8, LPS, NF-κB, PM, TLR, Dairy Farms, PM, Inflammation

## Abstract

**Background:**

The purpose of the present study was to investigate activation of inflammatory markers in human macrophages derived from the U937 cell line after exposure to particulate matter (PM) collected on dairy farms in California and to identify the most potent components of the PM.

**Methods:**

PM from different dairies were collected and tested to induce an inflammatory response determined by the expression of various pro-inflammatory genes, such as Interleukin (IL)-8, in U937 derived macrophages. Gel shift and luciferase reporter assays were performed to examine the activation of nuclear factor kappa-light-chain-enhancer of activated B cells (NF-κB) and Toll-like-receptor 4 (TLR4).

**Results:**

Macrophage exposure to PM derived from dairy farms significantly activated expression of pro-inflammatory genes, including IL-8, cyclooxygenase 2 and Tumor necrosis factor-alpha, which are hallmarks of inflammation. Acute phase proteins, such as serum amyloid A and IL-6, were also significantly upregulated in macrophages treated with PM from dairies. Coarse PM fractions demonstrated more pro-inflammatory activity on an equal-dose basis than fine PM. Urban PM collected from the same region as the dairy farms was associated with a lower concentration of endotoxin and produced significantly less IL-8 expression compared to PM collected on the dairy farms.

**Conclusion:**

The present study provides evidence that the endotoxin components of the particles collected on dairies play a major role in mediating an inflammatory response through activation of TLR4 and NF-κB signaling.

## Background

Inhalation of particulate matter (PM) and bioaerosol exposure, specifically from agricultural settings, has been shown to have a negative impact on the respiratory system of individuals and animals. Dairies are a large contributor to agriculture revenues in California, with little known about worker exposure to PM and bioaerosol. Exposure to dust on dairy farms may induce systemic reactions, increased bronchial responsiveness and chronic respiratory symptoms; all of which are frequently observed in farm workers [1].

Dairy farmers are exposed to organic dusts of a complex nature, and chronic respiratory symptoms are frequently observed in dairy farm workers. PM from dairies contain toxic and immunogenic constituents including histamine, endotoxins, mite antigen, cow urine antigen and microrganisms [2]. The bacterial content includes whole bacteria and cell wall components, such as endotoxin, lipopolysaccharide (LPS) derived from Gram negative bacteria and peptidoglycan, which is the main cell wall constituent of Gram positive bacteria. These substances are known to be biologically active, and some can induce chronic airway inflammation [3].

Previous studies have elucidated the initiation of inflammatory events following exposure to PM from dairies or organic dust [4-6]. Exposure to endotoxin has been associated with increased respiratory symptoms in occupational settings and described as a risk factor for organic dust toxic syndrome [7], but studies also suggest that endotoxin exposure may protect agricultural workers from allergic disease [8]. Recent reports indicate that workers without a farm childhood had an increased risk of allergic sensitization (defined by a positive response to allergens) and the development asthma (a chronic inflammatory disorder of the airways) compared to a population with an early life exposure to farming [8,9]. Furthermore, two cross-sectional studies have shown, that children who lived on farms had a lower risk to develop asthma than the children in the reference group [10]. From their results, the authors concluded that the exposure to a wider range of microbes by growing up on a farm could explain part of the protective effect against asthma [10].

The inflammatory response is thought to be caused by bacteria and fungi present in the PM [11]. Along with being exposed to organic dust on dairies, agricultural workers in dry climate regions are also exposed to substantial concentrations of inorganic dusts from agricultural soils. This inorganic component is associated with increased small airway disease among California farm workers [12]. Although an association between chronic bronchitis and dust exposure has been found, asthma was only associated with keeping livestock but not with dust exposure [1]. This supports the inflammatory potential of PM from dairies.

The purpose of the present study was to explore the inflammatory effects of macrophage exposure to dairy PM that may be of relevance for the generation of health effects, a type of study that is difficult to perform in vivo. Our main aim was to investigate the activation of inflammatory markers in human U937 macrophages after exposure to PM collected on various dairy farms in California.

## Methods

### Reagents and PM collection

National Institute of Standards and Technology (NIST) Standard Reference Material (SRM) 1649, an atmospheric particulate material collected in an urban area, and a diesel exhaust particulate sample, NIST SRM 2975, were purchased from NIST (Gaithersburg, MD). [y-^32^P] ATP (6000 Ci/mmol) was purchased from ICN (Costa Mesa, CA). PM from dairy farms were collected with a high-volume air sampler (model GS2310; Andersen Instruments Inc., Smyrna, GA) equipped with a four-stage cascade impactor (series 230, Andersen Instruments, Inc.) in the summer months of the year 2008 from various dairy farms and an urban area (Fresno, CA) of the San Joaquin Valley in California. The dairies included in the study were modern style dairies that included freestall barns and flush lanes, with at least 2,000 lactating cows (dairy #51: 2620 total cows, #54: 3200 total cows, #56: 4950 total cows, #57: 9550 total cows, #60: 2400 total cows).

Slotted aluminum substrates (Tisch Environmental, Cleves, OH) were used for PM collection. The nominal flow rate used for collection was 20 ft^3^/min, with particle size cutoffs of 10.2, 4.2, 2.1, and 1.3 μm, and these four fractions were named fraction A, B, C and D, respectively. Coarse PM is referred to as particles with a mass median aerodynamic diameter range of 10.2-2.1 μm and fine PM as particles within 2.1-1.3 μm. After collection, substrates from each stage were weighed; particles were removed by scraping with a spatula and stored at -80°C in vials. Stock solutions of particles were prepared before use by suspending them in autoclaved distilled water and by ultrasonication for 2 min at maximum power (100 W). Particles were used at 1, 5, or 10 μg/ml culture medium. 2,3,7,8-Tetrachlorodibenzo-*p*-dioxin (TCDD, > 99% purity) was originally obtained from Dow Chemicals Co. (Midland, MI). Dimethylsulfoxide (Me_2_SO), Phorbol-12-myristate-13-acetate (TPA), polymyxin B (PMB), Tumor Necrosis Factor (TNF)-α and lipopolysaccharide (LPS, Escherichia coli O55:B5) were obtained from SIGMA (St. Louis, MO). Other molecular biological reagents were purchased from Qiagen (Valencia, CA) and Roche (Indianapolis, IN).

### Endotoxin analysis

Samples were extracted by vortexing the filter in a TWEEN (10 ml at 0.05%) solution with pyrogen free water for 1 hour at 20-22°C and analyzed for biologically active endotoxin using the recombinant factor C assay (rFC) according to the manufacturer (Lonza Inc., Walkersville, MD), which detects activation of Factor C utilizing a fluorogenic according to Saito et al. [13]. The samples, 100 μl of blank, and an endotoxin standard (Escherichia coli 055:B5) were added to a 96 well plate. The plates were then pre-incubated for 10 minutes at 37°C. A mixture of 100 μl of rFC enzyme solution, buffer, and fluorogenic substrate at a 1:4:5 ratio were then added. The plates were incubated for 1 hour at 37°C. Fluorescence was read at time 0 and 1 hour in a fluorescence microtiter plate reader (Biotek Instruments, Winooski, VT). Excitation was read at 380 nm and emission at 440 nm. To obtain the endotoxin concentration, the log difference in fluorescence (RFU) was plotted against the log endotoxin concentration (EU/ml) in a linear regression curve. The standards used linear axis and polynomial regression curve, degree 2. Blanks taken in the field and plate well blanks along with spiking assays were used for quality control in order to account for any field contaminates and lab factors affecting fluorescence, such as pyrogen free water, reagent water centrifuge tubes pipette tips and microplates. Dilution of some samples was necessary; in these cases, a 50-fold dilution was performed.

### Cell culture and transient transfection

We obtained human U937 monocytic cells from the American Tissue Culture Collection (Manassas, VA) and maintained them in RPMI 1640 medium containing 10% fetal bovine serum (Gemini, Woodland, CA), 100 U penicillin, and 100 μg/ml streptomycin supplemented with 4.5 g/L glucose, 1 mM sodium pyruvate, and 10 mM HEPES. Cell culture was maintained at a cell concentration between 2 × 10^5 ^and 2 × 10^6 ^cells/ml. For differentiation into macrophages, U937 cells were treated with TPA (5 μg/ml) and allowed to adhere for 48 hr in a 5% CO_2 _tissue culture incubator at 37°C, after which they were fed with TPA-free medium.

For transient transfection of U937 macrophages, luciferase reporter constructs were transfected via Nucleofector technology as described preiviously [14]. Briefly, 10^6 ^U937 macrophages were resuspended in 100 μl Nucleofector Solution V (Amaxa GmbH, Köln, Germany) and nucleofected with 1.0 μg plasmid DNA using program V-001, which is preprogrammed into the Nucleofector device (Amaxa GmbH). Following nucleofection, the cells were immediately mixed with 500 μl of prewarmed RPMI 1640 medium and transferred into six-well plates containing 1.5 ml RPMI 1640 medium per well. After 24 h transfection, macrophages were treated with PM or LPS (control) for 4 h. Luciferase activities were measured with the Luciferase Reporter Assay System (Promega, Madison, MI) using a luminometer (Berthold Lumat LB 9501/16, Pittsburgh, PA). Relative light units are normalized to β-galactosidase activity and to protein concentration using Bradford dye assay (Bio-Rad, Hercules, CA).

### Cell viability assay

To assess the effect of PM on the viability of U937 macrophages, a trypan blue exclusion test was used [15]. A 10-μl portion of re-suspended cell pellet was placed in 190 μL PBS with 200 μl trypan blue (0.5% dilution in 0.85% NaCl). After a 5 min, 10 μl of the cell suspension was loaded into a hemocytometer, and the proportion of nonviable to viable cells was determined.

### Quantitative real-time reverse transcription-PCR

Total RNA was isolated from U937 cells using a high-pure RNA isolation kit (Roche), and cDNA synthesis was done as previously described [16]. Quantitative detection of β-actin and differentially expressed genes was performed with a LightCycler Instrument (Roche Diagnostics, Mannheim, Germany) using the QuantiTect SYBR Green PCR Kit (Qiagen) according to the manufacturer's instructions. DNA-free total RNA (1.0 μg) was reverse-transcribed using 4 U Omniscript reverse transcriptase (RT; Qiagen) and 1 μg oligo(dT)_15 _in a final volume of 40 μl. The primers for each gene (Table 1) were designed on the basis of the respective cDNA or mRNA sequences using OLIGO primer analysis software provided by Steve Rozen and the Whitehead Institute/MIT Center for Genome Research [17] so that the targets were 100-200 bp in length. PCR amplification was carried out in a total volume of 20 μl containing 2 μl cDNA, 10 μl 2 × QuantiTect SYBR Green PCR Master Mix, and 0.2 μM of each primer. The PCR cycling conditions were 95°C for 15 min followed by 40 cycles of 94°C for 15 sec, 60°C for 20 sec, and 72°C for 10 sec. Detection of the fluorescent product was performed at the end of the 72°C extension period. Negative controls were concomitantly run to confirm that the samples were not cross-contaminated. A sample with DNase- and RNase-free water instead of RNA was concomitantly examined for each of the reaction units described above. To confirm the amplification specificity, the PCR products were subjected to melting curve analysis. All PCR assays were performed in triplicate. The intra-assay variability was < 7%. Data was analyzed with the LightCycler analysis software.

**Table 1:** Primers for quantitative real time PCR analyses

Gene	Forward primer (5' - 3')	Reverse primer (5' - 3')
β-actin	GGACTTCGAGCAAGAGATGG	AGCACTGTGTTGGCGTACAG

COX-2	TGAAACCCACTCCAAACACA	GAGAAGGCTTCCCAGCTTTT

CYP1a1	TAGACACTGATCTGGCTGCAG	GGGAAGGCTCCATCAGCATC

IL-6	GAACTCCTTCTCCACAAGCG	TTTTCTGCCAGTGCCTCTTT

IL-8	CTGCGCCAACACAGAAATTA	ATTGCATCTGGCAACCCTAC

TNFα	CAGAGGGAAGAGTTCCCCAG	CCTTGGTCTGGTAGGAGACG

SAA1	GCCGATGTAATTGGCTTCTC	AGCCGAAGCTTCTTTTCGTT

### Gel-mobility-shift assay (GMSA)

Nuclear extracts were isolated from U937 cells, as described previously [16]. In brief, 5 × 10^6 ^cells were treated with PM or LPS for 90 min unless noted otherwise in the figure legends, and harvested in Dulbecco's PBS containing 1 mM PMSF and 0.05 μg/μl of aprotinin. After centrifugation, the cell pellets were gently resuspended in 1 ml of hypotonic buffer (20 mM HEPES, 20 mM NaF, 1 mM Na_3_VO_4_, 1 mM Na_4_P_2_O_7_, 1 mM EDTA, 1 mM EGTA, 0.5 mM PMSF, 0.13 μM okadaic acid, 1 mM dithiothreitol, pH 7.9, and 1 μg/ml each leupeptin, aprotinin, and pepstatin). The cells were allowed to swell on ice for 15 min and then homogenized by 25 strokes of a Dounce-homogenizer. After centrifugation for 1 min at 16,000 × *g*, nuclear pellets were resuspended in 300 μl ice-cold high-salt buffer (hypotonic buffer with 420 mM NaCl, and 20% glycerol). The samples were passed through a 21-gauge needle and stirred for 30 min at 4°C. The nuclear lysates were microcentrifuged at 16,000 × *g *for 20 min, aliquoted and stored at -80°C. DNA-protein binding reactions were carried out in a total volume of 15 μl containing 10 μg nuclear protein, 60,000 cpm of DNA oligonucleotide, 25 mM Tris buffer (pH 7.5), 50 mM NaCl, 1 mM EDTA, 0.5 mM dithiothreitol, 5% glycerol, and 1 μg poly (dI-dC). The samples were incubated at room temperature for 20 min. Competition experiments were performed in the presence of a 100-fold molar excess of unlabeled DNA fragments. Protein-DNA complexes were resolved on a 4% nondenaturating polyacrylamide gel and visualized by exposure of the dehydrated gels to X-ray films. For quantitative analysis, respective bands were quantified using a ChemiImager™4400 (Alpha Innotech Corporation, San Leandro, CA).

### Statistical analysis

All experiments were repeated a minimum of three times, and data are expressed as mean ± SD. Differences were considered significant for P < 0.05. Comparison of two groups was made with an unpaired, two-tailed student's *t*-test. Comparison of multiple groups was made with ANOVA followed by Dunnett or Tukey test.

## Results

### Dose-dependent effect of PM from dairy farms on inflammatory factors

To address the dose-dependent effect of PM, the mRNA expression of pro-inflammatory genes 6 hr after treatment with various concentrations of PM was studied. As shown in Table 2, treatment of U937 macrophages for 6 h with the fine particles of PM fraction C with a cutoff of 2.1 μm from dairy #57 in the range of 1, 5, or 10 μg/ml cell culture medium led to a dose-dependent mRNA induction of cyclooxygenase 2 (COX-2), Tumor necrosis factor-alpha (TNF-α), IL-6, IL-8, and serum amyloid A (SAA1). In addition, COX-2, TNF-α, IL-6, and IL-8 mRNA expression was significantly increased by PM C compared to control at the low concentration of 1 μg/ml. In contrast, SAA1 was significantly induced only at 5 or 10 μg/ml fine PM. The most conspicuous effect of fine PM from dairy #57 was found in the cases of COX-2, TNF-α and IL-8 (over 35-fold increase), followed by IL-6 and SAA1 expression.

**Table 2:** Dose-dependent effect of dairy PM on COX-2, TNF-α, IL-6, IL-8, and SAA1 mRNA expression compared to the dose-dependent effect of LPS.

Gene	PM (μg/ml)			LPS (μg/ml)		
		
	1	5	10	0.1	0.5	1.0
COX-2	10.6 ± 0.2^a^	15.9 ± 1.4^a^	43.5 ± 2.4^a^	14.5 ± 1.3^a^	48.2 ± 2.4^a^	82.1 ± 4.1^a^

TNF-α	8.1 ± 0.3^a^	19.8 ± 0.6^a^	42.5 ± 1.2^a^	9.4 ± 1.4^a^	16.8 ± 1.4^a^	42.2 ± 2.6^a^

IL-6	3.2 ± 0.4^a^	7.5 ± 0.4^a^	22.1 ± 0.7^a^	6.2 ± 0.4^a^	14.2 ± 1.0^a^	25.2 ± 1.1^a^

IL-8	9.3 ± 0.4^a^	17.4 ± 2.1^a^	35.6 ± 1.7^a^	12.8 ± 1.4^a^	22.5 ± 1.3^a^	51.6 ± 1.3^a^

SAA1	1.6 ± 0.4	2.7 ± 0.4^a^	4.6 ± 0.3^a^	2.0 ± 0.2^a^	3.2 ± 0.3^a^	7.8 ± 1.2^a^

U937 macrophages were treated with 1.0, 5.0, or 10.0 μg/ml PM C (size cutoff: 2.1 μm) from dairy farm #57 for 6 h. As a positive control, cells were treated with 0.1, 0.5, or 1.0 μg/ml LPS for 6 hours and mRNA was analyzed by real time RT-PCR. Control cells received 1% PBS. Results are normalized to β-actin and given as fold increase of the mRNA levels in treated cells versus controls (= 1). Values are given as mean ± SD of triplicates of three independent experiments

^a^significantly increased compared to control cells (p < 0.05)

To estimate the toxic potency, the effects of fine PM were compared with LPS, which has been shown to be an efficient inducer of inflammatory factors in U937 macrophages. As shown in Table 2, a concentration-dependent increase of COX-2, TNFα, IL-6, IL-8, and SAA1 mRNA expression showing a 14.5-, 9.4-, 6.2-, 12.8-, and 2.0-fold increase, respectively, at the lowest concentration (0.1 μg/ml) of LPS tested was observed. Time-dependent analysis of the mRNA increase of the inflammatory markers after PM exposure showed a maximum increase as early as 6 h after initial treatment, which sustained over a period of 24 h (data not shown). Therefore, U937 macrophages were treated for 6 h to analyze mRNA expression of the target genes.

### Effect of different size fractions of PM collected from various dairy farms on IL-8 expression

**Figure 1:**
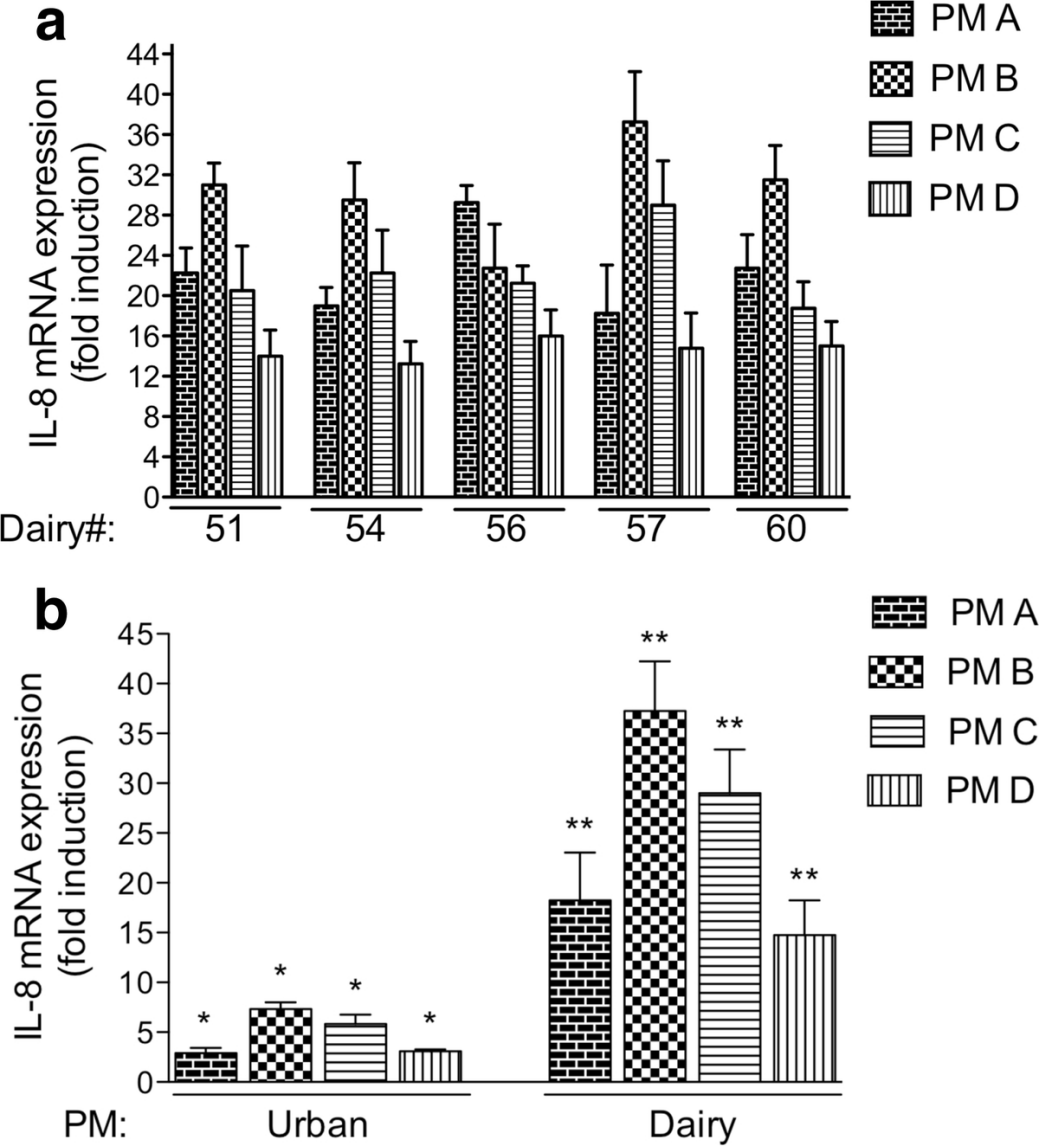
**A) Effect of PM collected on five different dairy farms in California (#51, 54, 56, 57, and 60) on the expression of IL-8 in human U937 macrophages**. Cells were treated with 10 μg/ml PM collected with a High-Vol sampler containing different particle size cutoffs of 10.2 (PM A), 4.2 (PM B), 2.1 (PM C), and 1.3 μm (PM D) for 6 h. IL-8 mRNA expression was determined using real-time PCR. Values are given as mean ± SD of three independent experiments and expressed as fold increase above control cells. **B) **Effect of PM collected on dairy #57 in California compared to PM collected in an urban area (Fresno, CA). Cells were treated for 6 h with 10 μg/ml PM collected with a High-Vol sampler containing particle size cutoffs of 10.2 (PM A), 4.2 (PM B), 2.1 (PM C), and 1.3 μm (PM D) or treated with 10 μg/ml of PM collected in a neighboring urban area (Fresno, CA). IL-8 mRNA expression was determined using real-time PCR. Values are given as mean ± SD. *Significantly increased compared to control cells (*p *< 0.05); **Significantly increased compared to cells treated with urban PM (*p *< 0.05).

The potency of PM collected from different dairies to induce an inflammatory response determined by the expression of IL-8 was tested. The pro-inflammatory marker IL-8 was selected for the mechanistic studies since IL-8 has been identified as a sensitive marker for inflammation and PM exposure [18,19]. PM with a size cutoff of 10.2 (fraction A), 4.2 (fraction B), 2.1 (fraction C), and 1.3 μm (fraction D) were collected from five different dairies in California. Both coarse (2.5-10 μm diameter) and fine (< 2.5 μm diameter) PM collected from the dairies had a significantly induced IL-8 mRNA in the human U937 macrophages (Figure 1A). PM from four out of the five dairies included in this study with a size cutoff of 4.2 μm tended to more actively induce IL-8 mRNA expression on an equal-dose basis than PM of 10.2, and 2.1, and 1.3 μm.

Treatment with 10 μg/ml coarse PM and fine PM collected from the California dairies caused a significantly stronger inflammatory response regarding the induction of IL-8 mRNA in the human U937 macrophages compared to 10 μg/ml PM collected from an urban area (Fresno, CA) in the same region as the dairies (Figure 1B). PM from dairy #57 were selected for mechanistic studies since dairy #57 represents a prototypical dairy of all five dairies investigated and is located in proximity to the Fresno area, where urban PM were collected for comparison. Urban PM with a size cut off of 4.2 and 2.1 μm (PM B and C) significantly induced IL-8 by 7- and 5-fold, respectively. Urban PM with a size cut off of 10.2 and 1.3 μm (PM A and D) induced IL-8 approximately 3-fold compared to control (Figure 1B).

### Effect of TLR4 and NF-κB inhibitors on PM-mediated induction of IL-8

**Figure 2:**
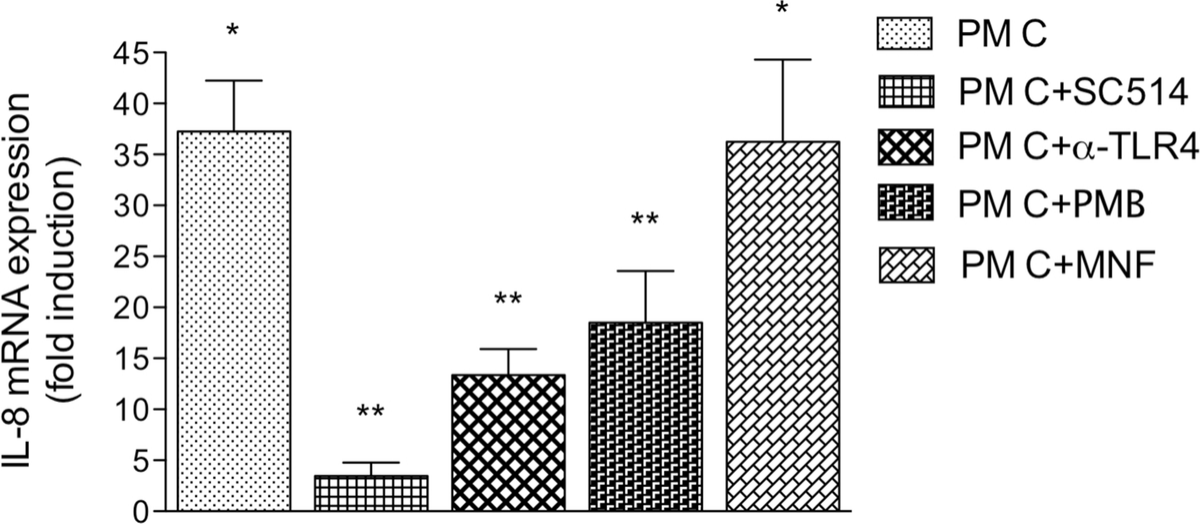
**Effect of various inhibitors on dairy PM-induced mRNA expression of IL-8**. To measure the ability to neutralize the TLR4-mediated PM response on U937 macrophages, 0.5 μg/ml of this TLR4 antibody (α-TLR4) was incubated with cells for 1 hour at 37°C. To inhibit activation of NF-κB or AhR, cells were pre-incubated with SC514 (5 μM) or MNF (5 μM) for 1 hr. To neutralize LPS cells were treated with 10 μg/ml Polymyxin B-treated PM C (PM C + PMB). Following pre-incubation, PM C from dairy #57 (10 μg/ml) was added. After 6 hr, cells were harvested to analyze IL-8 mRNA expression via real-time PCR and results were normalized to β-actin and given as fold increase compared to the mRNA level in control cells (= 1). Values are given as mean ± SD. *Significantly increased compared to control cells (*p *< 0.05); **Significantly lower than PM-treated cells (*p *< 0.05).

SC514 is a cell-permeable and selective IKK-2 inhibitor that blocks NF-κB-dependent gene expression. Pre-incubation of the U937 macrophages for 15 min with 5 μM SC514 blocked the induction of *IL-8 *mediated by fine PM (PM C) by approximately 70% (Figure 2). In order to neutralize the TLR4 pathway, U937 macrophages were incubated with an anti-hTLR4 antibody (5 μg/ml). After 1 h, the cells were treated with 10 μg/ml PM C from dairy #57 for 6 h. Pretreatment with anti-hTLR4 antibody blocked 65% of the PM-mediated induction of IL-8 (Figure 2). Furthermore, to neutralize the soluble fraction of endotoxin associated with the PM samples, we treated added Polymyxin B (PMB), an antibiotic that specifically binds LPS and prevents its binding to TLR4 receptors on the macrophages. PMB was added to the PM suspension at a concentration of 10 μg/mL and was pre-incubated for 30 min prior to addition of PM to the cells. PMB-treated PM C from dairy #57 effectively inhibited the induction of IL-8 by more than 50% compared to the untreated PM. To test whether PM collected from dairies contained components that activate the Aryl hydrocarbon receptor (AhR), as shown for diesel PM from heavy-duty vehicles [19], the AhR antagonist MNF, which blocks ligand-dependent activation of the AhR, was used. The AhR is a cytosolic ligand-dependent transcription factor that translocates into the nucleus and binds the xenobiotic response element (XRE) located on the promoter of cytochrome P450 (CYP)1A1 gene. As shown in Figure 2, MNF had no significant effect on PM-mediated IL-8 induction in contrast to SC-514, PMB, or the anti-hTLR4 antibody.

### PM from dairy farms induce NF-κB DNA-binding and NF-κB reporter activity

**Figure 3:**
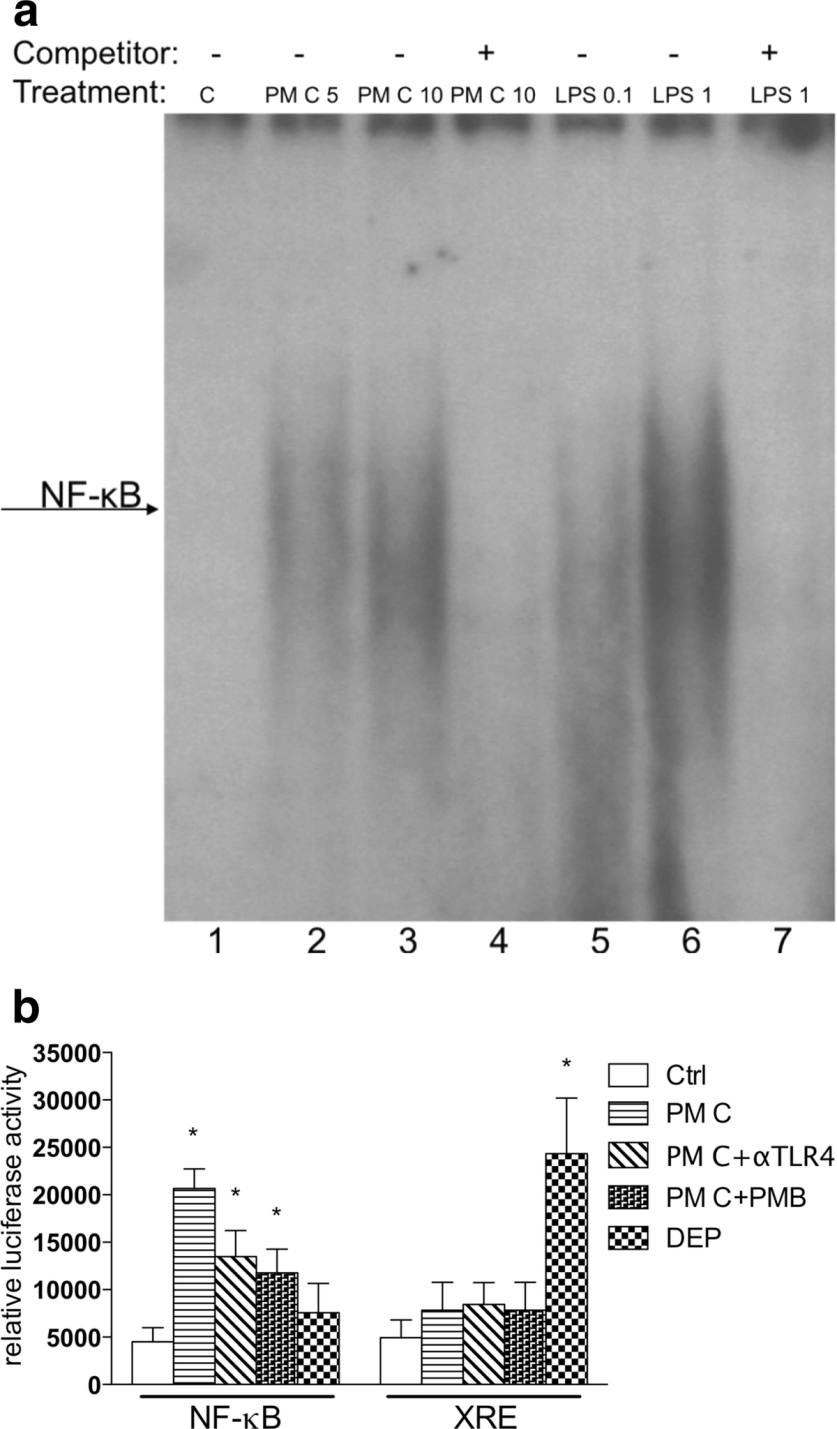
**Activation of NF-κB after PM exposure**. Exposure to PM from dairy farms stimulates NF-κB activity. **A) **Results from a gel shift assay illustrate the increased DNA-binding activity of NF-κB. U937 human macrophages were exposed for 1 h to 5 μg/ml and 10 μg/ml PM C (cutoff: 2.1 μm) collected on dairy farm #57. As a positive control, cells were exposed to pure endotoxin E.coli LPS (0.1 and 1 μg/ml LPS). The arrow indicates the formation of activated NF-κB complex binding to the NF-κB consensus DNA sequence. **B) **Activation of a NF-κB reporter plasmid after exposure to 10 μg/ml PM fraction C from dairy farm #57 in the absence or presence of a TLR4 neutralizing antibody (αTLR4) or 10 μg/ml Polymyxin B-treated PM C (PM C + PMB). In parallel, U937 macrophages were treated with particles from diesel engine exhaust (DEP) as a positive control for xenobiotic response element (XRE) reporter activity. Cells were transiently transfected with NF-κB or XRE luciferase reporter constructs and treated for 4 h with 10 μg/ml PM C or DEP.

Activation of TLR4 is known to activate the NF-κB signaling pathway. Possible NF-κB complex binding to a NF-κB consensus element after treatment of U937 macrophages with dairy PM was investigated to determine if induction of IL-8 by PM is associated with NF-κB activation. U937 macrophages were treated for 1 h with 5 or 10 μg/ml PM C (2.1 μm) collected on dairy farm #57. Results show that both concentrations of PM C enhanced NF-κB binding compared to unexposed control cells (Figure 3A). Ten μg/ml PM C (lane 3) led to a higher increase of NF-κB binding than 5 μg/ml PM C (lane 2). As expected, LPS (positive control), significantly and dose-dependently enhanced DNA-binding activity to the NF-κB consensus element (lane 5 and 6). A 100-fold excess of cold NF-κB oligonucleotide completely abolished formation of PM C- or LPS-stimulated NF-κB complexes (Figure 3A, lanes 4 and 7).

In addition to GMSA, NF-κB reporter activity was investigated after PM exposure. As shown in Figure 3B, exposure of U937 macrophages to 10 μg/ml PM C from dairy #57 for 4 h significantly increased NF-κB luciferase reporter activity approximately 4-fold compared to control cells. Suppression of TLR4 with a neutralizing TLR4 antibody and the LPS-neutralizing antibiotic PMB decreased the PM-mediated reporter activity by about 35%. The effect of PM on XRE reporter activity was studied in order to test if PM from dairies would activate the AhR signaling pathway. Results in Figure 3B show that only diesel engine exhaust particles (DEP, 10 μg/ml) but not PM from dairy farm #57 were able to induce the XRE reporter activity. DEP are well known to contain polycyclic aromatic hydrocarbons (PAH), which can bind to and activate the AhR and induce XRE reporter activity and CYP1A1 gene expression [19].

### Endotoxin units in PM

**Figure 4:**
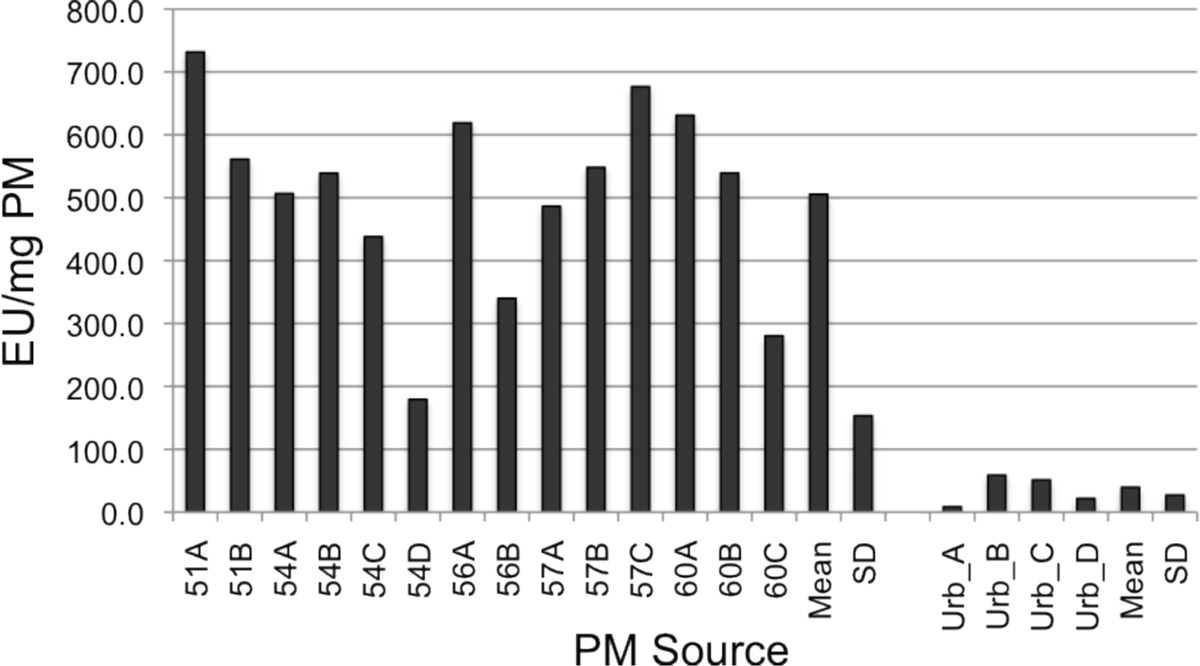
**Abundance of endotoxin in PM collected on dairy farms in California**. The concentration of endotoxin in PM from various size fractions collected on five different dairies (51, 54, 56, 57, and 60) and an urban area in Fresno, CA (urb) was analyzed via rFC. Values are shown as endotoxin units (EU) per mg PM mass.

Previous studies, including our own, have found that PM collected from dairies are loaded with endotoxin [11]. In order to test the concentration of endotoxin, PM from five different dairies were collected, and the amount of endotoxin was compared to PM collected in an urban area of Fresno, CA. Due to the limited amount of PM available, only certain size fractions could be analyzed. The rFC analysis revealed an average of about 510 EU/mg including all PM fractions collected on five different dairy farms. With the exception of dairy #57, the endotoxin concentration tended to be higher in coarse PM fractions with a size cutoff of 10.2 (PM A) and 4.2 μm (PM B) than in fine PM with a cutoff of 2.1 (PM C) and 1.3 μm (PM D) as shown in Figure 4. The PM collected in an urban area in Fresno, CA, contained an approximate mean of 40 EU/mg PM with the highest concentration of approximately 59 EU/mg in the coarse PM fraction. Fine particles within 1.3 μm contained significantly less endotoxin.

## Discussion

Recent studies report that PM collected from urban pollution or diesel and gasoline engines contribute to cardiovascular morbidity and mortality and may induce inflammatory responses in vitro as well as in vivo, including in humans [20]. However, data from experiments investigating the effects of PM from agricultural facilities and dairies are scarce. The present study shows that treatment of human macrophages with PM collected from California dairies leads to increased expression of pro-inflammatory marker genes, such as IL-8, TNF-α, and COX-2, and upregulates acute phase proteins IL-6 and SAA1, which increase in concentration following infection, inflammation, or trauma.

Cells of the innate immune system, such as macrophages, recognize pathogens via pattern recognition receptors (PRR), such as TLR [21]. In LPS-sensitive U937 cells, LPS from E. coli is an agonist of TLR4. In this study, this led to a dose-dependent induction of the abovementioned inflammatory marker genes in the U937 macrophages. In addition, induction of the pro-inflammatory genes after exposure to LPS was associated with activation of NF-κB. The organic dust from a dairy farm contains microbial constituents that emanate from Gram positive as well as Gram negative bacteria, and gene activation may, therefore, be caused by the combined engagement of different TLRs.

To study the mechanism of the PM-induced inflammatory response, we selected IL-8 as a hallmark of inflammation. IL-8 has been demonstrated in chronic diseases including chronic obstructive pulmonary disease (COPD) [22,23] and ulcerative cholitis [24]. We have found that the receptor of IL-8 (CXCL2) is involved in atherogenesis induced by environmental pollutants, such as dioxin [25], indicating the importance of IL-8 in the development of pathological endpoints.

The effect of ambient particles collected from an urban area (Fresno, CA) located in the San Joaquin Valley were significantly less potent in inducing IL-8 than PM from dairies, which contained 10-fold higher concentrations of endotoxin than the urban PM. Coarse PM fractions from the dairies with a cut off of 10.2 and 4.2 μm (PM A and PM B) contained more endotoxin than the fine fractions (PM C and PM D), which tended to correlate with a higher induction of IL-8 by PM A and B, although the effect was not statistically significant.

PM-activated gene expression of the inflammatory markers in this study was significantly suppressed by SC514, an inhibitor that blocks activation of NF-κB. This result indicates involvement of the NF-κB signaling pathway to mediate the effect of PM from dairies. Furthermore, exposure to dairy PM clearly activated NF-κB binding activity in the U937 macrophages. Addition of a TLR4 antibody to the culture medium before treatment with dairy PM neutralized approximately 60% of the PM-mediated effect on activation of NF-κB activity and the expression of IL-8. These data indicate that part of the PM components act through the TLR4 and that endotoxin is likely to be a critical component in the PM collected from dairies. The results also suggest that dairy PM-induced inflammation, such as IL-8 activation, is not entirely dependent on endotoxin and may include other components of dairy PM as perhaps double-stranded RNA or DNA of viruses, which may activate other TLR isoforms. The assumption, that other components in addition to endotoxin are triggering the inflammatory response, are also supported by the calculation of the endotoxin content in the dairy PM. Most dairy PM contained about 500 EU/mg which corresponds to 50 ng/mg. Accordingly, the exposure to 10 μg PM/ml would reflect an exposure to 0.5 ng endotoxin/ml, however, the inflammatory response is still stronger compared to 100 ng LPS/ml. Interestingly, Poole et al. showed that agricultural PM may mediate their inflammatory response also by activating the TLR2 pathway [26], CD14 mediated responses [27] and effects of specific components of dairy PM such as muramic acid [28].

Treatment with LPS and activation of TLR4 is well known to activate NF-κB [29]. Thus, activation of NF-κB by PM treatment is likely to be mediated through the activation of TLR4, which is supported by the result that the addition of neutralizing TLR4 antibody inhibited approximately 35% of the PM-mediated activation of NF-κB in the luciferase reporter assay. In addition to the results from the GMSA and inhibitor studies, these data suggest that induction of IL-8, including other inflammatory genes, is mediated not only via TLR4, but also via NF-κB signaling. These results underline the involvement of NF-κB and indicate the supportive action of NF-κB on PM-mediated transcriptional activation of pro-inflammatory genes.

In contrast to PM derived from diesel engine exhaust (DEP), PM collected from dairies did not activate AhR regulated XRE activity, which is known to be activated by PM generated from traffic and combustion processes [19]. In contrast to NF-κB or TLR4, the classical AhR-XRE pathway is mostly responsible for rapid responses to xenobiotics and activation of genes encoding xenobiotic metabolizing enzymes, including CYP1A1, through XRE binding sites located on the promoter of the gene. More recently, studies have focused on investigating the toxicity of agricultural dust particles and human health effects. Animal excrement generates a large part of the pollution on dairies, and some facilities may create a sizeable amount of road dust from vehicular traffic on gravel and unpaved roads [11].

In summary, the results from this study indicate that the most critical component of dairy farm dust is endotoxin, which may trigger local and systemic inflammatory reactions upon inhalation. In addition to endotoxin, allergens, microbial pathogens, bacterial toxins, fungal spores, and mycotoxins can attach to dust particles that, when inhaled, have the potential to cause local and systemic inflammatory reactions.

## Conclusions

In conclusion, exposure to PM collected on dairy farms generates an inflammatory response in human macrophages partly mediated through activation of TLR4 and the NF-κB signaling cascade. The inflammatory response induced by the urban PM was significantly lower compared to PM from dairies, which has a higher concentration of endotoxin than the urban PM.

## Abbreviations

AhR: Aryl hydrocarbon receptor; COPD: Chronic obstructive pulmonary disease; CAFO: Concentrated animal feeding operations; DEP: Diesel engine exhaust; IL-8: Interleukin 8; LPS: Lipopolysaccharide; NIST: National Institute of Standards and Technology; NF-kB: Nuclear factor kappa-light-chain-enhancer of activated B; PM: Particulate Matter; SRM: Standard Reference Material; TLR: Toll like receptor; XRE: Xenobiotic response element.

## Competing interests

The authors declare that they have no competing interests.

## Authors' contributions

CV designed and performed experiments, analyzed the results and wrote the manuscript. JG helped with LPS analysis and writing the manuscript. DW, DA, AL, and PW performed experiments and assisted with data interpretation. YZ and NK managed PM collection and analysis. DB, MS, and FM designed study, providing overview of all the steps of sample collection, and helped writing the manuscript. All authors read and approved the final manuscript.
